# Metastatic Pulmonary Calcification with Coexisting Non-specific Interstitial Pneumonia: A Rare Case Report and Literature Review

**DOI:** 10.7759/cureus.4183

**Published:** 2019-03-06

**Authors:** Arati A Inamdar, Rajiv Pulinthanathu

**Affiliations:** 1 Department of Pathology, RWJBarnabas Health, Livingston, USA; 2 Department of Pathology, Saint Barnabas Medical Center, Livingston, USA

**Keywords:** metastatic pulmonary calcification, non specific interstitial pneumonia, chronic kidney disease

## Abstract

Patients with underlying chronic kidney disease (CKD) often have elevated serum calcium and parathyroid hormones due to compromised kidney function. We present a case of a 63-year-old female non-smoker with a surgical history of three renal transplants (at age 47, 51, and 58) along with thyroidectomy and parathyroidectomy, who came to the emergency department with complaints of a persistent dry cough and shortness of breath for the last two months. The patient had been on immunosuppressive drugs-tacrolimus, prednisolone, and mycophenolic acid-since her first renal transplant as well as on cinacalcet after parathyroidectomy (at age 54). An initial computed tomography (CT) scan demonstrated ground glass opacities in the bilateral upper lobes while bronchoscopy revealed few inflammatory cells without any fungi or bacteria. A repeat CT scan performed five days later due to rapid progression of her clinical symptoms showed worsening of ground glass opacities in the bilateral upper lobes and new nodules in the right middle and lower lung lobes. A wedge lung biopsy revealed metastatic pulmonary calcification (MPC) in the right upper lobe and non-specific interstitial pneumonia (NSIP) in the right lower lobe, confirming the co-existence of two different pathological processes most likely complicating the patient's clinical symptoms. Despite comprehensive medical therapy, the patient's symptoms progressively worsened and she is currently undergoing evaluation for both renal and lung transplants. Our case report not only presents a rare case of MPC coexisting with NSIP but also sheds light on the associated morbidity due to pulmonary symptoms in CKD patients.

## Introduction

Respiratory diseases constitute a major cause of morbidity and mortality worldwide. Lung diseases can be caused due to primary pathology to lung parenchyma or due to pathology involving other organ systems such as renal, cardiovascular, and gastrointestinal [1]. Patients with chronic kidney disease (CKD) have impaired pulmonary function as a result of circulating uremic toxins and/or due to fluid overload, anemia, immune suppression, extra-osseous calcification, malnutrition, electrolyte disorders, and/or acid-base imbalances [2]. Especially, calcium imbalance often predominates in some patients leading to deposition of calcium-phosphate products in alveolar epithelial basement membranes over a period of time. Such calcium deposition in lung parenchyma, though rare, is known as metastatic pulmonary calcification [3]. Hemodialysis as well as renal transplant often aid in improving the pulmonary symptoms and metabolic derangements in CKD patients, thus preventing such complications [4].

Interstitial lung diseases (ILD) are a heterogeneous group of primary lung disorders characterized by alveolar septal thickening, fibroblast proliferation, and collagen deposition, which culminate in pulmonary fibrosis. Interstitial lung diseases can be classified into four groups: (1) ILD of known causes; (2) idiopathic interstitial pneumonias (IIP); (3) granulomatous ILD; and (4) ILD with well-defined clinicopathologic features [5]. Non-specific interstitial lung disease (NSIP) is one of the rare histological subtypes of idiopathic interstitial pneumonia characterized by the uniform appearance of interstitial inflammation mainly with lymphocytes and rarely with plasma cells. Two patterns of NSIP are described: cellular pattern and fibrosing pattern [6].

We hereby present the case of a 63-year-old female non-smoker who despite having three renal transplants and parathyroidectomy developed severe persistent cough and shortness of breath. A computer tomography (CT) scan without contrast revealed worsening of bilateral ground glass opacities. A biopsy from the upper lobe was consistent with metastatic pulmonary calcification (MPC), histologically showing the calcification in the interstitial compartment without significant scarring or architectural distortion. In addition, the biopsy from the lower lobe showed chronic interstitial inflammation with focal fibrosis and mild peri-bronchiolar metaplasia favoring the diagnosis of NSIP of cellular variant type. MPC has never been reported to co-exist with other pulmonary conditions and, to our knowledge, this case represents a rare combination of MPC and NSIP at the two lobes of the right lung. The exact etiological agent for NSIP in our patient could be exposure to a drug, most likely, tacrolimus. We suspect that the combination of these two pathological rare entities could be responsible for the on-going worsening of the patient's clinical course. Our case report also underscores the importance of prompt diagnosis via biopsy of the lung parenchyma in resistant or rapidly progressing cases. This approach not only helps in providing the effective targeted therapies but also is valuable in assessing the disease prognosis.

## Case presentation

**Figure 1:**
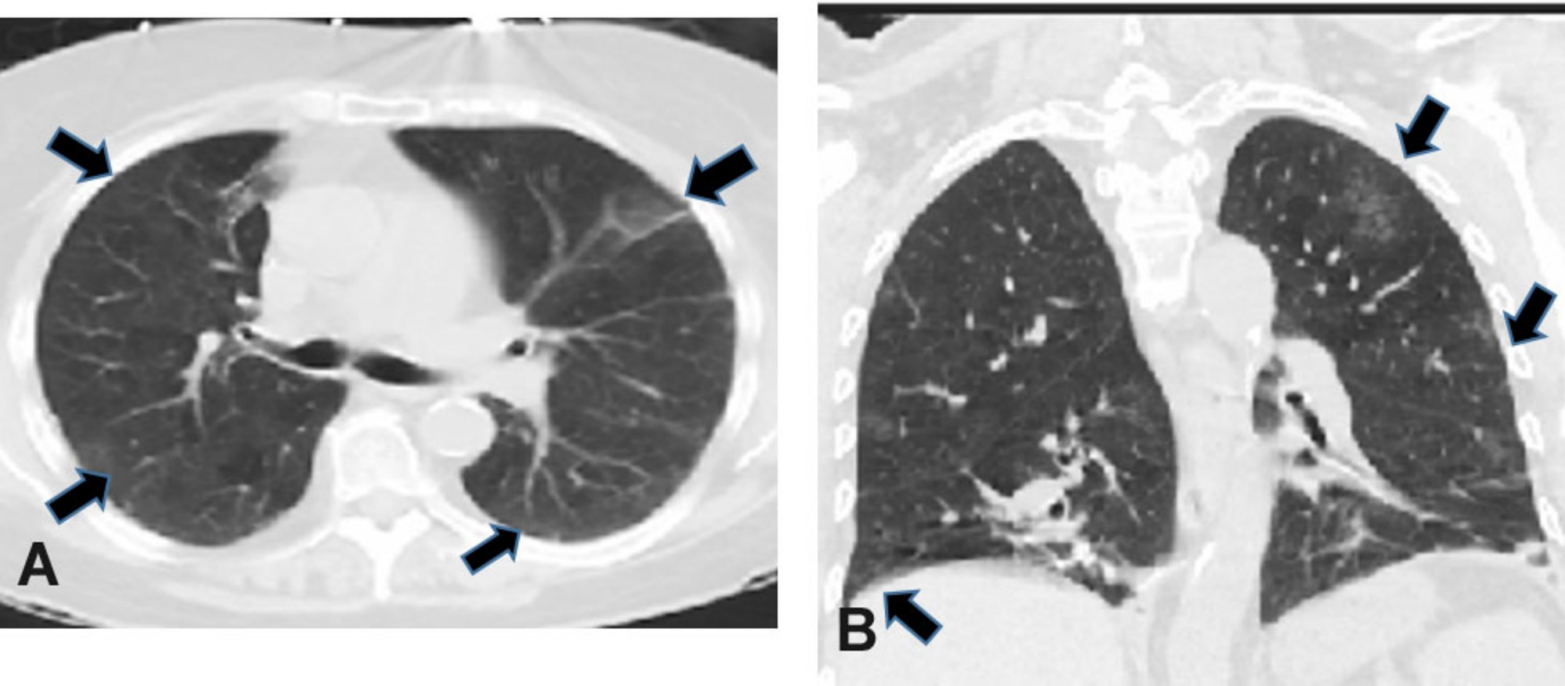
Computer tomography scan images of lungs. A, Axial CT with bilateral ground glass opacities (arrows) in the right and left upper lung lobes. B, Coronal CT with bilateral ground glass opacities (arrows) in the right and left upper and lower lung lobes.

A 63-year-old female non-smoker with a medical history significant for hypertension, obesity, chronic kidney disease, surgical history of three renal transplants (at age 47, 51, and 58 due to acute renal failure following abortive pregnancy at the age of 44 years), thyroidectomy, and parathyroidectomy presented to the emergency department with a persistent cough and shortness of breath for the past two months. She was without any fever, night sweats, weight loss, chills, hemoptysis, or hematemesis. The admission laboratory blood work revealed the following: estimated glomerular filtration rate (eGFR) 28.24 ml/min (normal (N):> 60 ml/min), blood urea nitrogen (BUN) level 49.2 mg/dl (N:10-26 mg/dl), creatinine 1.81 (N:0.4-1.1 mg/dl), serum calcium 12.4 (N:8.4-10.5 mg/dl), hemoglobin (Hb) 10 g/dl (N:12-15.4 g/dl), hematocrit (Hct) 30.4 (N:35-46.9%), and neutrophils 83.2% (N:42.0-76.0%). The home medication list included immunosuppressive agents tacrolimus, prednisolone, mycophenolic acid, cinacalcet, and carvedilol. The chest X-ray was non-significant; however, the admission CT scan revealed multiple focal areas of ground-glass opacities throughout both lung fields, predominantly in the upper lobes and a 3 mm sub-pleural nodule in the right middle lobe. The patient was diagnosed with pneumonia and prescribed antibiotics. Five days later the patient complained of worsening of cough and dyspnea. A repeat CT scan showed diffuse bilateral ground-glass densities in the right and left upper and lower lobes along with the presence of a newly developed 3 mm sub-pleural nodule in the right lower lobe (Figures 1A, 1B).

**Figure 2:**
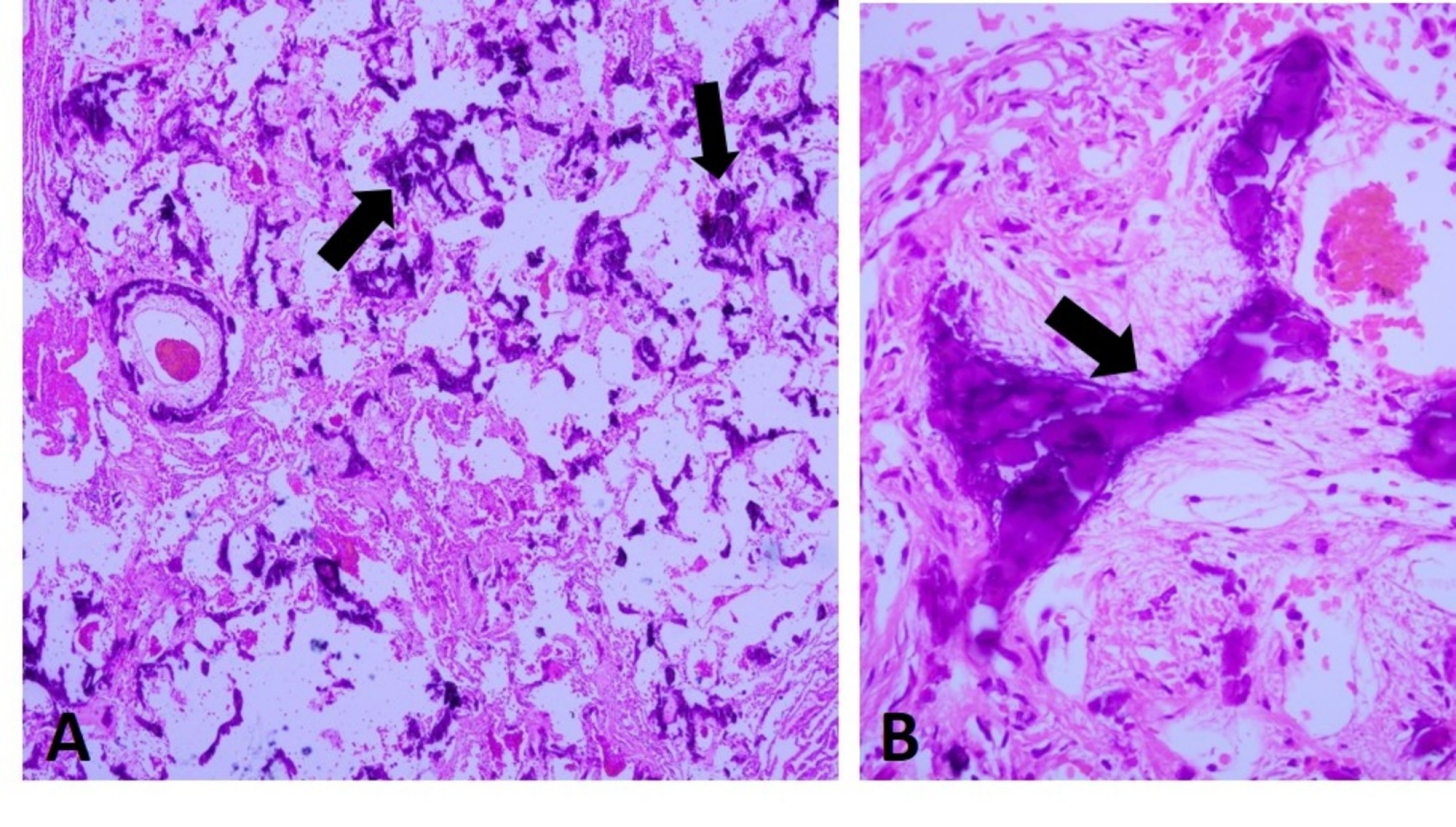
Metastatic pulmonary calcification (MPC) in the right upper lung lobe. A, Low-power view of areas of extensive MPC (hematoxylin-eosin, original magnification x 40). B, High power view of calcified area (arrow) (hematoxylin-eosin, original magnification x 400).

The pulmonary function test revealed mild restrictive lung disease with reduced vital capacity (67%) and moderately reduced diffusion capacity (46%). Bronchoscopy revealed inflammatory cells in the bronchial lavage. Considering the on-going rapid progression in the patient's symptoms, two weeks later, right video-assisted thoracoscopic surgery was performed to obtain a wedge biopsy specimen from the right upper and lower lobes. The right upper lobe demonstrated extensive calcification in the interstitial compartment without significant scarring or architectural distortion, consistent with MPC (Figures 2A, 2B).

**Figure 3:**
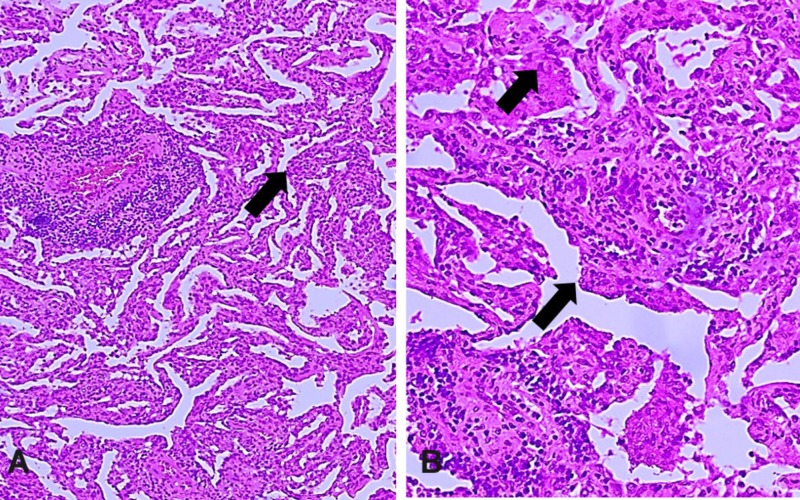
Non-specific interstitial pneumonia (NSIP) in the right lower lung lobe. A, Low-power view of areas of extensive NSIP (hematoxylin-eosin, original magnification x 40). B, High power view of affected area (arrows) (hematoxylin-eosin, original magnification x 400).

The right lower lobe showed chronic interstitial inflammation with focal fibrosis and mild peri-bronchiolar metaplasia favoring the diagnosis of non-specific interstitial pneumonia (NSIP) of cellular variant type (Figures 3A, 3B).

No eosinophils, pneumocystis jiroveci, or other fungal organisms were identified. The exact causative agent for NSIP was not clear in our patient but drug-induced NSIP could be considered.

## Discussion

MPC is a rare metabolic lung disease associated with end-stage renal disease and occurs due to derangement in the calcium metabolism, resulting in deposition of calcium-phosphate products in the alveolar epithelial basement membranes [3]. MPC is often asymptomatic and is rarely diagnosed ante mortem. It is often an autopsy finding in 60%-70% of patients with underlying renal failure [7]. MPC has also been reported in a variety of other benign conditions such as primary and secondary hyperparathyroidism, intravenous calcium therapy, osteoporosis, sarcoidosis, milk-alkali syndrome, Paget’s disease as well as with malignant conditions including parathyroid carcinoma, lymphoma, breast carcinoma, choriocarcinoma, malignant melanoma, synovial carcinoma, hypopharyngeal squamous cell carcinoma, massive osteolysis from metastases and multiple myeloma [8]. The metastatic calcium depositions are caused by the release of excess calcium salts from bone and their transport through the circulatory system to the organs. Interestingly, metastatic calcification can occur with normal or even low serum calcium levels; however, elevated calcium levels favor the formation of calcium-phosphate. High pH also leads to the formation of calcium phosphate salts and hence their deposition [9]. In addition to lungs, metastatic calcification has also been reported within the lamina propria of the stomach, in tubules and the interstitium of the kidneys [10]. The lung is especially susceptible to the formation of metastatic calcification as the pH of the blood in the lung is more alkalotic compared with other organs because of the active CO_2_ removal process at the lung apex and upper lobe as compared to other lung regions [9].

Despite having three renal transplants, our patient's admission laboratory workup revealed hypercalcemia and compromised renal function with baseline serum creatinine 1.4-1.5 mg/dl. Our patient was initially diagnosed with pneumonia based on the clinical and radiological presentation. MPC is often misdiagnosed as fungal infection or pneumonia. MPC is to be highly suspected in patients with CKD and persistent pulmonary symptoms even if radiological imaging and pulmonary function tests are inconspicuous [11]. High resolution CT scan is a sensitive imaging technique to diagnose MPC; however, other pulmonary conditions may radiologically present with ground glass opacities [3]. MRI and technetium 99m labelled bone scanning radionuclides are emerging as other imaging options capable of detecting and characterizing the calcium accumulation in lung parenchyma in MPC or other metabolic disorders [12-13]. In our patient, CT imaging revealed the ground glass opacities especially in the upper lobe in both lungs along with the 3 mm nodule in the right middle lobe (Figures 1A, 1B). Microscopically, calcification seen in MPC is mainly located in the interstitium of the alveolar basement membrane but calcification in the alveolar capillary walls, bronchial walls, and, to a lesser extent, bronchioles and media of pulmonary arterioles have also been reported [11]. In our case, a hematoxylin and eosin stained section from the right upper lobe wedge lung biopsy specimen revealed granular calcification in the interstitium of the alveoli with preserved alveolar architecture (Figures 2A, 2B).

Patients with MPC are rarely symptomatic; but in symptomatic patients the goal is to normalize the calcium and phosphate levels, which often help in alleviating the symptoms. Biphosphonates, calcimimetics, parathyroidectomy and increase in the frequency of dialysis have shown success in some patients [3]. Renal transplantation in renal failure patients is another option but worsening of MPC has also been reported despite successful transplantation [14-15]. Our patient has been on cinacalcet and immunosuppressive drugs since her parathyroidectomy and first renal transplant. Overall, she had three renal transplantations and is currently undergoing regular hemodialysis. The extensive calcification seen in the lung mostly due to persistent hypercalcemia led to a symptomatic form of MPC in our patient.

Recently, co-existence of MPC with renal cell carcinoma (RCC) has been reported [8]. In our patient, MPC was found to co-exist with NSIP, an incidental microscopic finding, diagnosed in a right lower lung lobe wedge biopsy specimen (Figures 3A, 3B). Interstitial lung diseases are a heterogeneous group of primary lung disorders characterized by alveolar septal thickening, fibroblast proliferation, and collagen deposition, which culminate in pulmonary fibrosis. Interstitial lung diseases can be classified into four groups: (1) ILD of known causes; (2) idiopathic interstitial pneumonias (IIP); (3) granulomatous ILD; and (4) ILD with well-defined clinicopathologic features [5]. NSIP is one of the rare histological subtypes of idiopathic interstitial pneumonia characterized by the uniform appearance of interstitial inflammation mainly with lymphocytes and rarely with plasma cells. Two patterns of NSIP are described: cellular pattern and fibrosing pattern [6]. In our patient, the alveolar interstitium showed mild, diffuse, and chronic interstitial pneumonia with infiltrates of mononuclear inflammatory cells with minimal fibrosis and without architectural distortion. Such findings are consistent with cellular variant of NSIP [16].

Patients with NSIP usually present with cough (33%-91%) and dyspnea (68%-100%) and may show inspiratory crackles. Pulmonary function reveals a restrictive ventilatory defect with decreased diffusing capacity. Radiologically, it presents as diffuse ground glass opacities, especially in the lower lung lobes [17]. Our patient also presented with persistent cough and shortness of breath with extensive rhonchi in the right mid lung region. Radiologically, the ground glass opacities were restricted to the bilateral upper lobes, and the pulmonary function test revealed restrictive with moderately reduced diffusion capacity. Due to clinical and radiological similarities in the presentation of MPC and NSIP, it was difficult to pinpoint the exact cause of the signs and symptoms in our patient until the wedge lung biopsy specimen from the upper and lower lung lobes showed the co-existence of both MPC and NSIP in the right lung specimen. NSIP is often associated with connective tissue diseases, drug and allergen exposure and in some cases coined as idiopathic [18]. In the absence of connective tissue disease in our patient, drug-induced NSIP is one possible cause for the symptoms. So far, cyclophosphamide, bleomycin, carmustine, statin, everolimus, and a few others have been implicated in drug-induced NSIP/ILD. However, tacrolimus has also been shown to cause lung injury and NSIP-like changes [19]. Our patient has been on tacrolimus since her first renal transplant and it could be one of the possible causes for NSIP changes seen in our patient. Symptomatic patients with NSIP require corticosteroids and, in resistant cases, cytotoxic agents such as azathioprine, cyclophosphamide, cyclosporine, mycophenolate mofetil and even rituximab have shown benefit [20]. Due to progressive worsening in the respiratory symptoms despite continuous supplemental oxygen and being on immunosuppressive agents, our patient is currently seeking consultation on renal and lung transplantation options.

## Conclusions

Despite being a rare entity, metastatic pulmonary calcification requires consideration in cases presenting with persistent respiratory symptoms. Furthermore, MPC can exist with other pathological entities such as NSIP, which presents with similar radiological features. Prompt diagnosis through open biopsy helps in formulating targeted management strategies and also prevents fatal complications, notably respiratory failure.
